# Psychological distress and its associated factors among patients with chronic obstructive pulmonary disease in Hunan, China: a cross-sectional study

**DOI:** 10.1038/s41598-023-32408-8

**Published:** 2023-03-30

**Authors:** Chunyu Wang, Jin Yan, Chenjuan Ma

**Affiliations:** 1 Xiangya Nursing School, Central South University, Changsha, Hunan China; 2 Department of Nursing, The Third Xiangya Hospital of Central South University, No. 138 Tong Zipo Road, Changsha, 410000 Hunan China; 3 New York University Rory Meyers College of Nursing, New York, NY USA

**Keywords:** Psychology, Risk factors

## Abstract

Patients with chronic obstructive pulmonary disease (COPD) experience a high risk for psychological distress. Understanding what factors contributing to this risk is vital for developing effective interventions to address COPD-related psychological distress. To examine psychological distress and its associated factors in COPD patients in China. This is a cross-sectional study. Using cluster random sampling, 351 COPD patients participated in and completed a questionnaire survey from June 2021 to January 2022. Instruments used in this research included a self-designed social-demographic questionnaire, the Kessler Psychological Distress Scale (K10), the COPD Knowledge Question, the Type D Personality Scale (DS-14), the COPD Assessment Test (CAT), and modified Medical Research Council Dyspnea Score (mMRC). Multivariate linear regressions were used in the final analysis. Among 351 COPD patients, 307 (or 87.5%) had psychological distress. Our univariate analysis indicated that psychological distress scores were significantly associated with monthly household income (F = 2.861, P < 0.05), exercise frequency (F = 4.039, P < 0.01), type D personality (t = 5.843, P < 0.01), years with COPD (*r*_*s*_ = 0.156, P < 0.01), frequency of acute exacerbation (*r*_*s*_ = 0.114, P < 0.05), mMRC score (*r*_*s*_ = 0.301, P < 0.01), and CAT score (*r*_*s*_ = 0.415, P < 0.01). Our final multivariate linear regression showed that exercise frequency (coefficient = −1.012, P < 0.01) was an independent protective factor of psychological distress in COPD patients, while type D personality (coefficient = 3.463, P < 0.001), mMRC score (coefficient = 1.034, P < 0.001) and CAT score were independent risk factors (coefficient = .288, P < 0.001). No relationship was observed between psychological distress and knowledge of COPD. Psychological distress is commonly presented among COPD patients in China. Findings from this study suggest promoting and increasing frequency of exercise will be beneficial in reducing psychological distress among COPD patients. This study also highlights the importance of assessing personality type, dyspnea, and impact of COPD on daily living for preventing and managing psychological distress due to COPD. In addition, Given the high rate of psychological distress among COPD patients, policymakers should consider making mental health resources easily available and accessible to this vulnerable population.

## Introduction

Chronic Obstructive Pulmonary Disease (COPD) is a global public health concern. It is associated with high morbidity and mortality, as well as a heavy economic burden^1,2^. Characterized by limited airway ventilation, COPD is an irreversible respiratory disease, with its main clinical symptoms being dyspnea, cough, expectoration, etc.^3^ It is estimated that the global incidence of COPD is about 10%^4^. According to the 2019 Global Burden Disease (GBD) report^5^, COPD has the highest mortality rate, and it is the 3rd leading cause of death after cardiovascular disease and cancer. Each year, approximately 5 million people become disabled due to COPD and 1 million people die from it^4^.

In China, COPD is also a critical public health concern, which is highlighted in the Healthy China 2030 Plan^6^. This plan underscores tremendous COPD-related challenges the Chinese healthcare system and society face and calls for immediate national actions on the prevention and treatment of COPD and related health issues. The total number of people experiencing COPD in China is estimated to be about 100 million, with the prevalence of COPD being over 27% for adults aged 60 years and older^7^. The number of Chinese males with COPD is about 2.2 times the number of females with COPD. In addition, the annual total medical expenses per COPD patient is about 33–40% of the average household income in China^8^.

COPD not only directly affects a patient's physical health due to a decrease in pulmonary function, it may also cause psychological distress due to its long-term and progressive nature. Studies have found that COPD patients were more likely to suffer from psychological distress or other mental illnesses, compared with the general population^9^. In one study, researchers reported that the incidence rate of psychological distress was 42.9% in community-dwelling COPD patients and 64% in those experiencing acute exacerbation^10^. Furthermore, suffering from psychological distress over time could lead to more severe clinical symptoms, poorer sleep quality, lower self-management ability, and decreased quality of life^11,12^.

At the same time, research has suggested that psychological distress may be prevented or reduced with appropriate intervention. The first critical step in such intervention development is to identify factors, particularly modifiable factors, influencing the development of psychological distress among patients with COPD^13–16^. However, to date, there are few studies on the associated factors of psychological distress in patients with COPD around the world and even fewer in China. Therefore, this study aimed to describe the rate of psychological distress in patients with COPD and explore factors associated with psychological distress. Findings from this study will assist in improving both physical and mental health, increasing self-management ability, and promoting the prognosis and recovery of COPD patients.

## Methods

### Study design

This is an observational, retrospective study. It was conducted in the outpatient respiratory department in five large tertiary hospitals in Hengyang, Hunan, China. Data were collected on-site using questionnaires from June 2021 to January 2022. All patients with COPD who met the inclusion and exclusion criteria were invited to participate.

### Sampling, sample, and data collection

In this study, cluster random sampling was used for selecting participants from five districts of Hengyang, Hunan Province. A list of tertiary hospitals in each district was obtained first and then one hospital was selected from each district using the random number table approach, which resulted in a total of five tertiary hospitals in this study. Within each hospital, all patients who visited the outpatient respiratory department (in a stable period) during the study period were invited to participate and screened for eligibility.

To be eligible for this study, patients had to meet the following inclusion criteria: (a) were diagnosed with COPD according to the Global Initiative for Chronic Obstructive Lung Diseases (GOLD) guidelines and had definite airflow limitation with a post-bronchodilator forced expiratory volume in 1 s (FEV1)/forced vital capacity (FVC) < 70%; (b) the visit is an outpatients regular visit (in a stable period) in order to improve the patient's family self-care ability; (c) ages ≥ 18; (d) able to communicate and complete the questionnaire independently or under the guidance of the researcher; (e) be willing to participate. Patients were excluded if they (a) had acute exacerbated symptoms and hospitalization within the past month; (b) combined with other serious diseases, such as severe cardiovascular disease; (c) had severe mental illnesses or cognitive impairment; (d) had already participated in other respiratory intervention programs such as pulmonary rehabilitation program.

Written informed consent was obtained from the patients and/or their close relatives when necessary. All participants were informed that they could discontinue or withdraw from the study at any time for any reason. A total of 368 eligible patients agreed to participate and completed the survey questionnaires under the guidance of a trained research assistant. Of those completed questionnaires, 17 had 20% or more incomplete answers and thus were excluded from analysis. This resulted in a final sample of 351 participants in this study or an effective response rate of 95.3%.

### Measurement

A questionnaire was used for data collection, which included items/questions on patient information of psychological distress, type D personality, the knowledge of COPD, COPD-related health status, and other health conditions (e.g., Body Mass Index (BMI), chronic conditions, etc.). It also collected patient socio-demographics (e.g., age, gender, education) and life style data.

#### Psychological distress

Psychological distress was measured by the Kessler Psychological Distress Scale (K10) in this study. The K10 is a self-rating scale that briefly screens for non-specific mental health condition-related symptoms, such as anxiety and stress levels, experienced in the past four weeks. It has been used broadly in research on mental health internationally. The K10 has also been translated into Mandarin and adapted for the Chinese population. Research has shown good reliability and validity in various samples in China^17,18^. The K10 includes 10 items measuring anxiety and depressive symptoms and uses a five-value response option for each question: “1 = none of the time,” “2 = a little of the time,” “3 = some of the time,” “4 = most of the time,” and “5 = all of the time.” The total score of K10, therefore, ranges from 10 to 50 with higher scores indicating a lower risk for anxiety or depression. In this study, the Cronbach α for K10 is 0.92, which indicated an excellent reliability.

In this study, for a descriptive purpose, patients were categorized into psychological distress group (K10 score >  = 15) and no psychological distress group (K10 score < 15) as some researchers have suggested a score of 15 as a cutoff point^19–22^. For univariate and multivariate analysis, psychological distress was considered as a continuous variable.

#### Type D personality

Type D personality was assessed by the Type D Personality Scale (DS-14), which consists of two subscales: negative affectivity (NA, negative affectivity, 7 items) and social inhibition (SI, social inhibition, 7 items). Each item of the DS-14 is set on a 5-point Likert scale from 0 (totally inconsistent) to 4 (completely suitable). Scores on both subscales ranged from 0–28 and can be calculated as follows: NA (item 2, 4, 5, 7, 9, 12, and 13) and SI (item 1, 3, 6, 8, 10, 11, and 14). The Chinese version of the DS-14 scale has good reliability and validity. Researchers has reported the Cronbach α of the two sub-dimensions as 0.832 for NA and 0.720 for SI among Chinese population, respectively^23^. In this study, the Cronbach α was 0.92 for the whole scale 0.92 and 0.54 ~ 0.87 for the subscales.

#### Knowledge of COPD

Patients’ knowledge of COPD was measured using the Chronic Obstructive Pulmonary Disease Knowledge Question (COPD-Q)^24^. The COPD-Q consists of 13 items related to various aspects of COPD, including clinical manifestations, risk factors, drug use, whether oxygen therapy can be used, whether it can be prevented, and outcomes. Each item has three answer options, "yes," "no," or "don't know." Each correct answer (a positive answer to “yes” or a reverse answer to “no”) is assigned 1 point. Incorrect answers and an answer of “don’t know” is assigned 0 point. The total scores of the COPD-Q thus range from 0 to 13 points. The higher the score, the higher the patient's knowledge level of COPD. Previous research in China has reported a Cronbach's α of this questionnaire as 0.72^25^. In this study, the Cronbach's α for COPD-Q was 0.72.

#### Health conditions

##### COPD-related health status

COPD-related Quality of Life (QoL). The COPD Assessment Test (CAT) was used to measure the impact of COPD on a patient’s life^26,27^. The CAT includes eight items in total and measures the following areas: cough, expectoration, chest tightness, wheeze, daily activities, confidence, sleep, and energy. The severity of each item ranges from 0 to 5 points in order from mild to severe, and the total score of CAT ranges from 0 to 40 points. The higher the total score, the worse the patient’s quality of life. This scale has good reliability and validity and is also recommended by GOLD. The Cronbach's α of each item was 0.76–0.79^28^. In this study, we found a Cronbach's α of 0.88.

Dyspnea. The modified Medical Research Council Dyspnea Score (mMRC) was used to measure the degree of dyspnea. The mMRC was originally a self-rating scale developed by Fletcher in 1959^29^. This scale rates dyspnea from 0 to 4 points, indicating dyspnea from strenuous exercise to dyspnea at rest, and is the easiest indicator to assess dyspnea. A higher score indicates more severe dyspnea. mMRC has been widely used in research of chronic respiratory disease, and was recommended in the Chinese guidelines for the diagnosis and treatment of chronic obstructive pulmonary disease (2021 revision) for evaluating functional dyspnea^30^.

We also measured years with COPD and frequency of acute exacerbation (i.e., number of hospitalizations in the past one year). Both were described as continuous variables. Oxygen use was also measured and treated as a dichotomous variable (Yes/No).

##### Other health conditions

Other health conditions measured included BMI and chronic conditions. Based on the BMI score, patients were categorized into three groups, those with BMI < 18.4, those with BMI > 23.8, and those with BMI between. To measure the presence of chronic conditions, patients were asked “whether you have asthma, hypertension, diabetes, heart disease, or other chronic comorbidities?” This variable was treated as a dichotomous variable (Yes/No) in analysis to indicate the presence (or not) of any chronic conditions.

#### Socio-demographics and life style

Patient’s socio-demographic information was collected, including age (< 60, 60–79, ≥ 80), gender (male/female), education (primary school and lower, junior middle school, high school, college or university), marital status (single, married, divorced, widowed), and monthly household income (< 3000, 3000–8000, > 8000). These variables were all treated as categorical variables.

In addition, we included two measures to reflect the life style of the participants, including exercise frequency and smoking history which are known important factors associated with COPD.

### Statistical analysis

Statistical analyses were performed using SPSS software, version 26.0. All variables were distributed close to normal. Categorical variables were described using frequency and percentage. Continuous variables were described using mean and standard deviation (SD). Univariate analysis was first conducted, namely T-test and analysis of variation (ANOVA), to compare the differences in psychological distress total score by categorical variables. Pearson correlation analysis was used to assess the correlation between total score of psychological distress and continuous variables. Followed by multivariate linear regression including variables that were identified as being significant associated with the outcome (i.e., psychological distress). To test the heterogeneity of the results of multivariate linear regression, we conducted a subgroup analysis according to gender (only males) and age (from 60 to 79) due to the limited sample size of other groups of them. Significant differences were set as statistically significant at the level of P < 0.05.

### Ethical approval and consent to participate

The study was approved by the Institutional Review Board of Xiangya Nursing School of Central South University (E202194). All methods were performed in accordance with the relevant guidelines and regulations. Informed consent was obtained from all participants.

## Results

### Participants’ characteristic statistics

Table 1 described participant characteristics. On average, patients were 69.67 years old (SD, 11.13) with the majority (65.3%) aged between 60–76 years. The vast majority of the 351 COPD patients were male (85.2%) having a junior high school education or below (76.4%) and married (84.0%). Two in five of the patients reported a monthly household income of < 3000 RMB. As for their life style, 71.5% had a smoking history and 61.9% reported exercising once or twice a week.

**Table 1:** Variables summary of COPD patients (n = 351).

Variables	Numbers	Proportion (%)
Ages		
< 60	63	17.9
60–79	229	65.3
≥ 80	59	16.8
Gender		
Males	299	85.2
Females	52	14.8
Education		
Primary school and lower	133	37.9
Junior middle school	135	38.4
High middle school	56	16.0
College or university	27	7.7
Marital status		
Single	4	1.1
Married	295	84.1
Divorced	18	5.1
Widowed	34	9.7
Monthly household income (RMB)		
< 3000	141	40.2
3000–8000	155	44.1
> 8000	55	15.7
Smoking history		
Yes	251	71.5
No	100	28.5
Exercise frequency		
1–2/a week	217	61.9
3–4/a week	50	14.2
5–6/a week	32	9.1
7/a week	52	14.8
BMI (kg/m^2^)		
< 18.4	240	68.4
18.4–23.8	93	26.5
> 23.8	18	5.1
Comorbidities		
Yes	275	78.3
No	76	21.7
Oxygen use		
Yes	133	37.9
No	218	62.1
D type personality	351	25.0 (19.0)
Yes	306	87.2
No	45	12.8
Psychological distress		
Yes	307	87.5
No	44	12.5

		Mean (SD)
Years with COPD		9.09 ± 8.91
Frequent acute exacerbation		1.76 ± 2.36
mMRC		2.03 ± 1.22
CAT		22.41 ± 8.81
Knowledge of COPD		4.88 ± 2.87
Psychological distress		25.31 ± 8.32

Mean, median value; SD, standard deviation; mMRC, modified Medical Research Council Dyspnea scale; CAT, COPD assessment test; BMI, body mass index.

The total mean score of psychological distress in this sample was 25.31 (SD, 8.32). Nearly 9 out of 10 (87.5%) has a psychological distress score of 15 or higher, a score that has been used sometimes as a cutoff point for indicating presence of psychological distress. In this study, 87.2% of patients had type D personality. The mean score of knowledge of COPD was 4.88 (SD, 2.87).

For variables of health conditions, the mean score of mMRC was 2.03 (SD, 1.22), the mean score of CAT was 22.41 (SD, 8.81), the mean score of years with COPD was 9.09 (SD, 8.91) and the mean score of frequent acute exacerbation was 1.76 (SD, 2.36). The majority had a BMI score lower than 18.4 (68.4%), were with comorbidities (78.3%), and did not report oxygen use in daily life (62.1%).

### Relationship between psychological distress and associated factors

#### Univariate analysis results

The univariate analysis from T-test and ANOVA) showed that the total score of psychological distress significantly varied by monthly household income (F = 2.861, P < 0.05), exercise frequency (F = 4.039, P < 0.01) and type D personality (t = 5.843, P < 0.01). There was no significant difference between other categorical variables (gender, ages, educational level, marital status, BMI, smoking history, comorbidities and oxygen use) and the total score of psychological distress (P > 0.05). These results are presented in Table 2.

**Table 2:** Factors associated with psychological distress from univariate analysis.

Variables	Group	F/t	*P*
Gender	Males	−1.120	0.263
	Females		
Ages	< 60	0.420	0.658
	60–79		
	≥ 80		
Educational level	Primary school and lower	0.882	0.704
	Junior middle school		
	High middle school		
	College or university		
Marital status	Single	0.851	0.467
	Married		
	Divorced		
	Widowed		
Monthly household income (RMB)	< 3000	4.039	0.003*
	3000–8000		
	> 8000		
BMI (kg/m^2^)	< 18.4	0.117	0.890
	18.4–23.8		
	> 23.8		
Smoking history	Yes	0.435	0.664
	No		
Comorbidities	Yes	0.597	0.551
	No		
Oxygen use	Yes	0.741	0.459
	No		
Exercise frequency	1–2/a week	2.851	0.037*
	3–4/a week		
	5–6/a week		
	7/a week		
Type D personality	Yes	34.142	< 0.001*
	No		

*P < 0.05.

Table 3 shows the results from pearson correlation analysis regarding the relationship between psychological distress and continuous variables in this study. The total scores of psychological distress were positively correlated with years of having COPD (*r*_*s*_ = 0.156, P < 0.01), frequency of acute exacerbations (*r*_*s*_ = 0.114, P < 0.05), CAT score (*r*_*s*_ = 0.415, P < 0.01), and the mMRC score (*r*_*s*_ = 0.301, P < 0.01). Tables 2 and 3 presented details of their relationship.

**Table 3:** Correlation analysis of the relationships between psychological distress and continuous variables.

	1	2	3	4	5	6
1	1.000					
2	0.156**	1.000				
3	0.114*	0.158**	1.000			
4	0.301**	0.179**	0.296**	1.000		
5	0.415**	0.204**	0.142**	0.393**	1.000	
6	−0.034	−0.087	0.098	0.067	−0.031	1.000

*P < 0.05, **P < 0.01.

1 = Psychological distress, 2 = Year with COPD, 3 = Frequent acute exacerbation, 4 = mMRC, 5 = CAT, 6 = knowledge of COPD.

#### Results from a multivariate linear regression

Taking the total score of psychological distress (continuous variable) as the dependent variable, significant (P < 0.05) factors from univariate analysis (monthly household income, exercise frequency, type D personality, year with COPD, frequent acute exacerbation, mMRC score, and CAT score) as independent variables, multivariate linear regression is used for final analysis (Table 4 for independent variable assignments). The results showed that exercise frequency was an independent protective factor while mMRC score, CAT score and type D personality were independent risk factors (in Table 5). Besides, our subgroup analysis indicates similar significant relationships between psychological distress (outcome) and personality (Type D), mMRC, and CAT with small difference in the strength of the relationship (in Appendix 1 and 2).

**Table 4:** Variables assignments.

Factors	Assignments instruction
Monthly household income (RMB)	< 3000 = 1, 3000–8000 = 2, > 8000 = 3,
Exercise frequency	1–2/a week = 1, 3–4/a week = 2, 5–6/a week = 3, 7/a week = 4
Type D personality	Yes = 0, No = 1
Frequent acute exacerbation	Measured value
Year with COPD	Measured value
mMRC	Measured value
CAT	Measured value

**Table 5:** Factors associated with psychological distress in multivariate analysis.

Variables	Unadjusted		Adjusted Beta	t	*P*
	B	SE			
Constant	16.561	1.330	–	12.455	0.000
Exercise frequency	−1.012	0.347	−0.136	−2.917	0.004
Type D personality	3.463	0.800	0.206	4.329	0.000
mMRC	1.034	0.345	0.151	2.998	0.003
CAT	0.288	0.048	0.305	5.959	0.000

F = 29.700, P < 0.001, R = 0.506, R^2^ = 0.256.

## Discussion

Our study demonstrated that most patients with COPD have psychological distress, with an incidence rate of 87.5%. This result is slightly higher than some previous studies^31,32^, but there are also studies showing that the incidence of anxiety and depression in COPD outpatient clinics ranges from 10 to 80%^33^, which is roughly the same as the results of this study. In addition, our data provided novel insights into the independent risk and protective factors of COPD patients with psychological distress. The results of multivariate analysis showed that mMRC and CAT were independent risk factors, and exercise frequency was an independent protective factor for psychological distress in patients with COPD. Specifically, COPD patients with less frequent exercise, higher mMRC scores, and higher CAT scores had higher levels of psychological distress.

### Exercise frequency

Consistent with previous studies^34^, this study found that exercise frequency was an independent protective factor for psychological distress in patients with COPD. Patients who exercise less frequently have higher levels of psychological distress. Due to the gradually descending lung function of COPD patients, the contractility of lungs and thorax will be weakened, the airway will be blocked, and the gas in the alveoli will be difficult to release, resulting in poor breathing, which will aggravate negative emotions. A certain frequency of exercise is beneficial to reduce the level of lipid peroxidation in plasma, reduce the susceptibility to exogenous mutations, and increase the repair speed of DNA damage^35^. In addition, exercise is also beneficial to enhance cardiopulmonary function, increasing the contractility of respiratory muscles, and then improving the status of lung function^36^. A study also illustrated that maintaining sufficient activity is beneficial to disease prevention and life health^37^. Therefore, medical staff should advocate and advise COPD patients to take appropriate physical exercise, which is conducive to enhancing muscle endurance, improving lung function, improving quality of life, and further relieving their psychological distress.

### mMRC and CAT

Our results presented that mMRC and CAT are independent risk factors for psychological distress. The higher the mMRC and CAT scores, the higher the level of psychological distress. Interestingly, the reliability of CAT measurement is improved in our study compared to other studies, which indicates our results could better reflect the real situation of the research population^38^. The mMRC score indicates the severity of dyspnea, and the CAT score indicates the level of quality of life associated with symptoms such as cough, expectoration, sleep, and energy. These two associated factors are related to a certain extent, and will both affect the level of psychological distress of patients with COPD. COPD patients with worsening conditions will have a certain degree of symptoms such as cough, shortness of breath, and difficulty breathing, which will cause certain damage to the heart and lungs, and then influence their daily life. At the same time, due to the repeated attacks of the disease, the patient feels disappointed and distressed about the disease. Physiological pain and psychological pain will jointly lead to negative emotions such as despair, anxiety, and depression in patients. Repeated acute exacerbations caused symptoms such as dyspnea, which increased the serum levels of inflammatory cytokines TNF-α, IL-6, etc^39^. While the infiltration of a large number of inflammatory cells aggravated the inflammatory response of the respiratory tract. At the same time, changes in these inflammatory cytokines can stimulate the hypothalamic–pituitary–adrenal axis (HPA axis), resulting in a decrease in its activity, which in turn increases the content of cortisol in serum, leading to neuroendocrine disorders, resulting in psychological distress^40^. Psychological distress also acts inversely on acute exacerbation of COPD, forming a vicious circle^41^, which is consistent with the findings of Holm et al.^42^. Therefore, early detection and early diagnosis of the psychological distress associated with COPD patients is beneficial to the prognosis of the disease.

### Type D personality

Multivariate analysis found that type D personality was another important independent risk factor affecting psychological distress in COPD patients. Other studies have also confirmed that results for many diseases associated with depression^43^. Type D personality is characterized by a joint tendency to experience NA and SI^44^. Individuals with high NA commonly have feelings of discomfort, depression and worry^45^. Studies have also demonstrated that, compared with patients with non-type D personality, patients with type D personality have negative thoughts about things and are more susceptible to negative stimuli, resulting in a poorer ability to overcome or adapt to stressful events^46^. High SI individuals are vulnerable to feeling inhibited and unsafe in interactions with others because of fears of disapproval and rejection. These feelings related to type D personality make the individual vulnerable to general psychological distress/disorders (e.g. depression), decreased quality of life, and various diseases^47^. The results of a study found that type D personality was not significantly correlated with mortality in patients with COPD^48^. However, other researchers have found that Type D personality is associated with depressive symptoms^49^. Therefore, further research is needed on the relationship between psychological distress and type D personality in COPD patients.

### Other health conditions

68.4% of COPD patients had lower BMI and 78.3% were with comorbidities. These health conditions seem related to psychological distress. However, the results of univariate analysis showed these factors are not related to psychological distress, which is contrary to Zhang et al. ^50^. This disparity may be explained as follows: 1) limited sample size leads to an inapparent association; 2) the relationship between health conditions and psychological distress may be restricted by other factors; 3) when collecting data, the research population didn’t fill in measurements carefully, which might present fatigue effect or social desirability bias. Therefore, the relationship between BMI, comorbidities and psychological distress needs to be further explored.

### Limitation

First, this study is a cross-sectional study and cannot infer whether the relationship between the factors has changed over time, and therefore cannot draw a relevant causal relationship; Second, our study is limited to COPD patients in Hunan Province, and may not apply to populations in other provinces and cities. Therefore, it is necessary to conduct a large sample and multi-center survey, which is conducive to reflecting the representativeness and generalization of the results; Third, the measurement tools in this study are all subjective scales, lacking objective indicators; Fourth, the use of measurement of psychological distress was completed by the patients over one month period, which contributes to recall bias.

## Conclusion

In conclusion, 87.5% of patients with COPD have psychological distress. The results showed that mMRC, CAT and type D personality were independent risk factors, and exercise frequency was an independent protective factor for psychological distress in patients with COPD. Given the high rate of psychological distress among COPD patients, policymakers should consider making mental health resources easily available and accessible to this vulnerable population. Prospective studies are also needed to investigate whether treatments that reduce symptoms and/or symptom variability may impact the long-term outcomes of patients with COPD.

## Supplementary Information

Supplementary Information.

## Data Availability

We are not making data available for open source as we haven’t sought consent (to make data available for open source) from the study participants while data collection. The corresponding author should be contacted if someone wants to request the data from this study.

## Abbreviations

COPD: Chronic obstructive pulmonary disease
K10: The Kessler Psychological distress scale
COPD-Q: The COPD Knowledge Question
DS-14: The type D personality scale
CAT: The COPD assessment test
mMRC: Modified Medical Research Council Dyspnea Score
GOLD: Chronic obstructive lung diseases
BMI: Body mass index
NA: Negative affectivity
SI: Social inhibition
SD: Standard deviation
ANOVA: Analysis of variation
