# Supplementary material for: Psychological distress and its associated factors among patients with chronic obstructive pulmonary disease in Hunan, China: a cross-sectional study
Source: Sci Rep. 2023 Mar 30;13:5199. doi: 10.1038/s41598-023-32408-8 (PMC10063563; doi:10.1038/s41598-023-32408-8)
Supplement: Supplementary file 1 — Supplementary Information. [file 41598_2023_32408_MOESM1_ESM.docx]

Appendix

Appendix 1. Subgroup analysis of factors associated with psychological distress in multivariate analysis (Males)

| Variables | Unadjusted | | Adjusted  Beta | t | *P* |
| --- | --- | --- | --- | --- | --- |
|  | B | SE |  |  |  |
| Constant | 16.780 | 1.371 | - | 12.238 | .000 |
| Exercise frequency | -.871 | .321 | -.139 | -2.715 | .007 |
| Type D personality | 3.351 | 0.859 | .202 | 3.902 | .000 |
| mMRC | .864 | .366 | .131 | 2.359 | .019 |
| CAT | .278 | .052 | .302 | 5.389 | .000 |

Note: F = 23.926, P＜0.001, R = 0.496, R^2^ = 0.246

Appendix 2. Subgroup analysis of factors associated with psychological distress in multivariate analysis (COPD patients aged from 60-79)

| Variables | Unadjusted | | Adjusted  Beta | t | *P* |
| --- | --- | --- | --- | --- | --- |
|  | B | SE |  |  |  |
| Constant | 14.977 | 1.455 | - | 10.291 | .000 |
| Type D personality | 3.560 | 1.008 | .212 | 3.532 | .000 |
| mMRC | .920 | .431 | .132 | 2.133 | .034 |
| CAT | .283 | .060 | .297 | 4.735 | .000 |

Note: F = 20.404, P＜0.001, R = 0.458, R^2^ = 0.209
